# From Mother–Fetus Dyad to Mother–Milk–Infant Triad: Sex Differences in Macronutrient Composition of Breast Milk

**DOI:** 10.3390/nu17091422

**Published:** 2025-04-23

**Authors:** Maria Lithoxopoulou, Calliope Karastogiannidou, Anastasia Karagkiozi, Iliani Eleni Zafeiriadou, Ekaterini Pilati, Elisavet Diamanti, Stavros Kalogiannis, Emilia Vassilopoulou

**Affiliations:** 1 2nd Department of Neonatology & NICU, School of Medicine, Faculty of Health Sciences, Aristotle University of Thessaloniki, Papageorgiou General Hospital, 56403 Thessaloniki, Greece; mlithoxopoulou@yahoo.com; 2 Department of Nutritional Sciences and Dietetics, School of Health Sciences, International Hellenic University, 57400 Thessaloniki, Greece; karasto@ihu.gr (C.K.); ilianazafeiriadou@gmail.com (I.E.Z.); kathrine.pilati@gmail.com (E.P.); vassilopoulouemilia@gmail.com (E.V.); 3 1st Department of Obstetrics and Gynecology, School of Medicine, Faculty of Health Sciences, Aristotle University of Thessaloniki, Papageorgiou General Hospital, 56403 Thessaloniki, Greece; ankaragkiozi@yahoo.com; 4 Pediatric Area, Fondazione IRCCS Ca’ Granda-Ospedale Maggiore Policlinico, 20122 Milan, Italy; 5 Department of Clinical Sciences and Community Health, University of Milan, 20120 Milan, Italy; 6 Department of Life Sciences, School of Life and Health Sciences, Universiy of Nicosia, Nicosia 2417, Cyprus

**Keywords:** breast milk, sex, colostrum, transitional breast milk, mature breast milk, fat, protein, non-protein nitrogen, total solids, energy

## Abstract

**Background:** The composition of breast milk is influenced by a variety of factors, including maternal anthropometric characteristics, dietary and lifestyle habits, and lactation and feeding parameters. Emerging evidence also suggests that an infant’s sex may play a role in shaping breast milk composition. **Objective**: This study aims to investigate the macronutrient composition of early breast milk up to 3 months postpartum, with a particular focus on potential differences related to the infant’s sex. **Methods:** A total of 102 breast milk samples were collected at four time points across three lactation stages, representing colostrum, transitional, and mature milk, from a cohort consisting of 51 consenting mothers of Mediterranean origin who met the inclusion criteria. The samples were analyzed using mid-infrared spectroscopy to determine their macronutrient composition. **Results:** Colostrum from mothers of male infants contained approximately 60% higher levels of nitrogenous compounds, crude protein, true protein, and non-protein nitrogen compared to colostrum from mothers of female infants. Transitional milk for female infants contained higher fat, total solids, and energy. No significant differences in macronutrient composition were observed in mature milk between the sexes. For both sexes, colostrum contained more nitrogenous compounds and fewer total solids than mature milk. Male colostrum had lower carbohydrate levels compared to the later stages of lactation, while female samples showed no significant changes. Strong positive correlations between fat and crude protein were found for both sexes during the transitional and mature milk stages. In male colostrum, all macronutrients were interrelated. **Conclusions:** The observed differences in the composition of colostrum and transitional breast milk for male and female neonates suggest potential sex-specific nutritional adaptations during early lactation. These findings may have implications for personalized infant nutrition strategies in cases where breast feeding is hampered, as well as for understanding early neonatal adaptations.

## 1. Introduction

Breast milk (BM) is the optimal nutrition for infants, providing a well-balanced composition of macronutrients and micronutrients tailored to their developmental needs. In addition to its nutritional value, BM contains bioactive components that contribute to meeting nutritional requirements, support immune system development, protect against pathogens, infections, gastrointestinal diseases, and chronic diseases, and enhance the diversity of the microbiome [1]. BM is highly complex and can vary significantly based on factors like maternal diet, lactation stage, race, regional environmental chemicals, and storage conditions.

Human milk oligosaccharides (HMOs) exhibit important immunological properties and prebiotic functions [2]. Over 400 different proteins in BM also possess enzymatic and other bioactive properties [3]. Essential fatty acids are present in the form of triglycerides, diglycerides, monoglycerides, phospholipids, and free fatty acids [4]. Bioactive agents in BM are provided to the infant, including growth factors that affect the intestinal tract, blood vessels, and nervous and endocrine systems, as well as hormones that regulate growth and metabolism and immune factors such as cells, immunoglobulins, and mucins [5]. Furthermore, microorganisms in BM contribute to infesting the newborn’s gastrointestinal tract with a healthy probiotic microbiome [6]. Moreover, the microRNAs (miRNAs) found in BM are small, single-stranded, non-coding RNA molecules that play a crucial role in immunoprotection and developmental programming [7].

The composition of both nutritive and non-nutritive components in BM varies based on multiple factors. Infant-related factors include birth weight, gestational age, infant age, and lactation stage. Maternal-related factors primarily involve diet and anthropometric characteristics, as well as pregnancy history and mode of delivery [8,9]. Time-related factors include lactation stage [6], feeding duration, breast emptying [10], and circadian variability [9]. Maternal diet strongly influences fatty acid composition. but also affects the macronutrient, micronutrient, and growth factor content of BM [8]. Fat content is affected by maternal diet, body weight during pregnancy and lactation, and feeding time [4].

Recent research suggests that infant sex is an additional factor influencing the composition of BM throughout lactation [9,11,12]. Bernardes-Loch et al. [13] reported that among 146 identified proteins in 98 BM samples, 42 varied in abundance between male and female infants. One study of 41 colostrum samples found that fat and energy were significantly higher in male infants than female infants, while the carbohydrate concentration was lower [12]. In contrast, another study analyzing 189 colostrum samples from healthy lactating mothers found no sex-based differences in macronutrient and energy content [14]. According to previous studies, it is known that males consume a greater amount of milk [15], however, this is not combined with the difference in macronutrient composition between the sexes. Some studies focus on differences in fat content and, thus, energy in male infants [16]. A study analyzing 135 samples of mature milk from healthy lactating mothers showed that the macronutrients and energy content in mature milk were not affected by the sex of the offspring [14]. In contrast, a study of Iranian women found that mature BM for female infants contained more fat and energy, potentially reflecting different metabolic needs between the sexes at different stages of lactation [11]. Although the mechanisms leading to these variations are still unclear, emerging evidence indicates sex differences both antenatally and postnatally [17].

Several studies focus on the mechanisms behind the variation in macronutrients according to sex. Early exposure to adiponectin and leptin through breast milk is thought to influence infant appetite regulation and metabolic programming [18,19,20]. A recent study found no consistent or biologically significant differences between male and female infants in milk leptin, insulin, and adiponectin concentrations [21]. The sex differences observed in one study [22] reflected an interaction between circulating sex hormones and milk-derived hormones. Additionally, human milk leptin and insulin may affect the development of the neonatal gut microbiome, which could influence gut inflammation and immune function [23], with potential implications for asthma development.

A large Finnish cohort study (STEPS) identified several maternal factors that influence the hormone and protein concentrations in milk, with sex-related interactions [24]. Milk glucose concentrations appear higher for male infants during the first month after birth. This difference in milk glucose may be more pronounced when mothers are overweight or obese, but these differences were not evident at 3 months postpartum [21].

Thus, this study aims to investigate the association between infant sex and BM macronutrient composition across three different lactation stages. The present report is the first in a series to provide a more in-depth analysis of BM composition, focusing on the impact of infant sex and lactation stage. Furthermore, the correlation between carbohydrate, fat, and crude protein levels in BM will be investigated.

## 2. Materials and Methods

### 2.1. Study Design and Population

The present cross-sectional observational study was conducted at the Papageorgiou General Hospital of Thessaloniki over a 24-month period, from November 2021 to October 2023. The study adhered to the ethical principles of the Declaration of Helsinki and was approved by the Papageorgiou Hospital Ethics Committee—Approval Code: 39438/19 November 2021. Informed consent was obtained from all participating mothers.

Milk samples were divided according to the sex of the offspring into male and female samples and were compared by mid-infrared spectroscopy with regard to their macronutrient contents.

A total of 51 consenting lactating mothers were recruited based on the following specific eligibility criteria: white Mediterranean origin, good health, full-term pregnancy, no medication use, no history of gestational diabetes, non-smoking, no alcohol or drug use, and willingness to donate breast milk as instructed by midwives, nurses, and doctors, For the study purposes, dietary data were collected within the first three months postpartum.

A complete medical record was also documented in a study entry which included the demographic and socioeconomic characteristics of both mothers and infants. This record covered details such as the infant’s sex, maternal ethnicity, age, weight, number and mode of delivery, and duration of breastfeeding for previous children, among other factors.

### 2.2. Breast Milk Collection and Analysis

Breast milk was collected from mothers who gave birth at the Papageorgiou General Hospital of Thessaloniki through finger expression or with a hospital-grade electric breast pump (Medela Symphony™; Medela Inc., McHenry, IL, USA), following the milk pumping instructions provided by the midwives and hospital medical staff. Samples were collected by electrical pumping, mostly from one breast (either right or left), separately from each lactating mother into sterile collection bottles or pumping bags at around noon (10:00 a.m.–2:00 p.m.) to minimize circadian variability in BM, one hour after feeding the baby ad libitum from one or both breasts until satisfied during breastfeeding.

Colostrum samples were collected between days 3 and 5 postpartum by manually expressing breast milk. Transitional milk samples were collected between days 9 and 12 postpartum, whereas mature milk samples were collected at 1 month and 3 months postpartum. However, the collection of all four samples at the different stages of lactation was not completed by all mothers, resulting in varying numbers of samples obtained at each sampling stage. After discharge from the hospital maternity department, the mothers collected their own milk at home and stored the samples in a domestic refrigerator until transportation to the laboratory, which occurred within 2–3 h in a portable cooler with ice packs. The samples were placed into Eppendorf tubes and stored in a professional laboratory freezer at −78 °C until they were analyzed using the MIRIS human milk analyzer.

**Figure 1:**
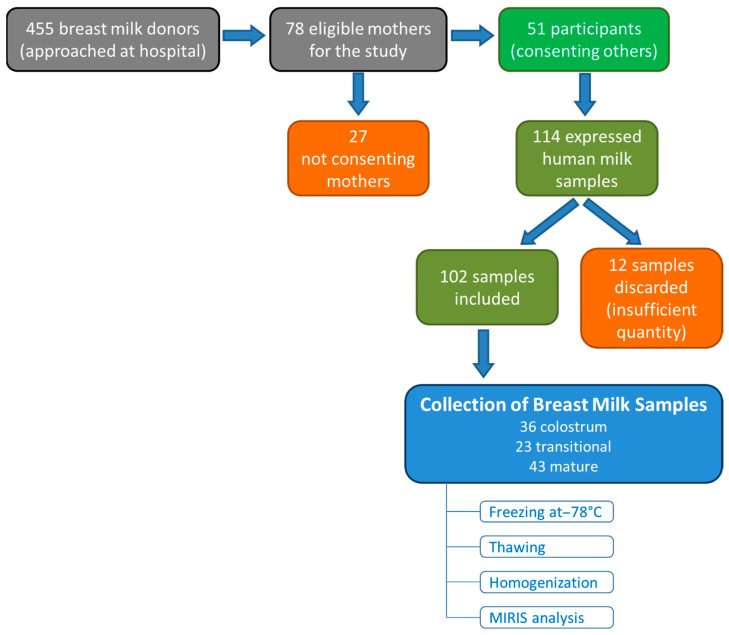
Participants and samples flow chart.

In total, 36 colostrum samples were collected, corresponding to 19 male and 17 female infants; 23 transitional milk samples were collected, corresponding to 12 male and 11 female infants; and 43 mature milk samples were collected, corresponding to 22 male and 21 female infants. In total, 102 samples of fresh maternal milk were collected across all three stages of lactation, with 53 samples from mothers of male infants and 49 samples from mothers of female infants. A flow chart of the mother recruitment and breast sample collection is depicted in Figure 1. Descriptive statistics of the demographic and clinical characteristics of the mothers and neonates in the colostrum, transitional milk, and mature milk groups are presented in Tables S1–S3 in the Supplementary Materials, respectively. The required sample volumes at different stages of lactation were 5 mL for colostrum and 10–20 mL for transitional and mature milk.

### 2.3. Analysis

The macronutrients (fat, carbohydrate, crude protein, true protein, and non-protein nitrogen) and energy contents of the ΒΜ samples were determined by employing mid-infrared spectroscopy. Milk samples were prepared and analyzed using the Miris Human Milk Analyzer (Miris HMA^®^, Miris AB, Uppsala, Sweden) according to the standardized operating procedures provided by the manufacturer. Calibration and quality control were performed prior to each measurement session to ensure the accuracy and reliability of the results. Aliquots of 3 mL were homogenized using the MIRIS ultrasonic processor and maintained at 40 °C prior to analysis.

### 2.4. Statistical Analysis

Normality tests were performed employing the Shapiro–Wilk test, considering the relatively low numbers of samples in all groups (*n* < 25). Pairwise group comparisons were performed using the Independent Samples *t*-test when both groups followed a normal distribution and the Mann–Whitney U test when at least one group did not follow a normal distribution. Differences in dispersion were statistically tested by analysis of variances using the F-test for normally distributed variables and the Levene’s test for all non-normally distributed variables. For multiple group comparisons, the Shapiro–Wilk normality test was used to assess normality, and the Levene test was used to test for equal variances. If all groups exhibited a normal distribution with unequal variances, multiple group comparisons were performed using Welch’s ANOVA and post hoc pairwise comparisons were performed using the Bonferroni post hoc test. When the *p*-values in the Shapiro–Wilk normality test were lower than 0.05, indicating a non-normal distribution, multiple group comparisons were performed using the non-parametric Kruskal–Wallis test and post hoc two-way comparisons were performed using the Dunn–Bonferroni method. All statistical analyses were implemented using an internet-based application, freely accessible at the URL https://statisty.app (accessed on 20 December 2024), except for the F-Test for Variances, which was performed using an application freely available at www.statskingdom.com. The statistical significance level was set at 0.05.

## 3. Results

The colostrum, transitional, and mature milk samples from mothers of male and female infants were compared in terms of crude and true protein, non-protein nitrogen (NPN), carbohydrates (CH), total solids (TS), and energy content, using either the Mann–Whitney U test or the Independent Samples *t*-test, according to the results of the normality tests. Although it was not possible to obtain samples from all the donors who initially volunteered to participate in the study, the cohort remained relatively unchanged throughout all sampling stages. Since the number of samples in all groups ranged between 11 and 23, a normality test was performed using the Shapiro–Wilk test, considering the relatively small number of samples. The normality tests revealed that only a limited number of variables followed a normal distribution, as shown in Table 1. Since a parametric method, such as the Independent Samples *t*-test, requires that both groups in the comparison need to follow a normal distribution, the only male–female comparisons made using the Independent Samples *t*-test were for carbohydrates in transitional BM and for fat and energy in mature BM. For all other sex-based comparisons, the Mann–Whitney U test was used. No outliers were excluded from the statistical analyses.

**Figure 2:**
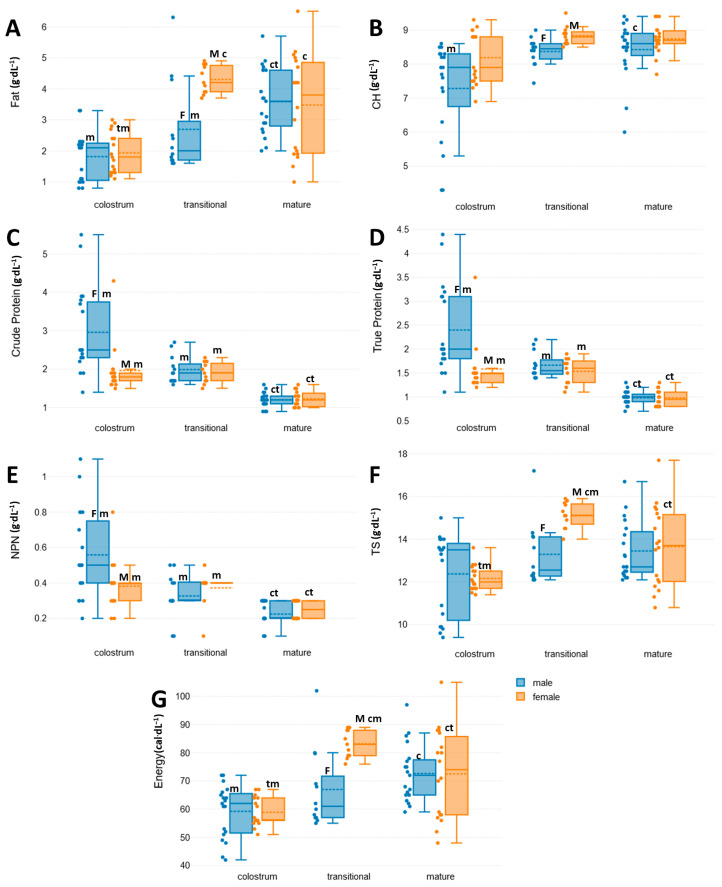
Box plots of the contents at the three stages of breast milk (colostrum, transitional, and mature breast milk) of female (orange) and male (blue) births: (**A**) crude protein, (**B**) true protein, (**C**) non-protein nitrogen (NPN), (**D**) fat, (**E**) carbohydrate (CH), (**F**) total solids (TS), and (**G**) energy. Indices signifying statistically significant differences: M, gender-specific difference to the male samples; F, gender-specific difference to the female samples; c, significant difference to the colostrum; t, significant difference to the transitional breast milk; m, significant difference to the mature breast milk.

As shown in Table 2, the exact *p*-values determined for crude protein, true protein, and NPN in colostrum were lower than 0.05, indicating statistically significant differences. All three variables exhibited higher mean values for male offspring, indicating that the protein levels in the colostrum samples from mothers of male neonates were higher. In particular, the average values for crude protein, true protein, and NPN were 2.96, 2.40, and 0.56 g/dL in colostrum for male infants and 1.97, 1.59, and 0.38 g/dL in that for female infants, respectively. Furthermore, the range of values obtained from the male samples was more widely dispersed compared to that of the female samples, which showed a greater uniformity, especially in colostrum. This difference is illustrated in Figure 2 and shown by the increased standard deviation (SD) and interquartile range (IQR) values in male samples compared to the respective values of the female colostrum samples. Specifically, the ratio of SD (Table 2 and Table 3) of male to female samples ranged from 1.8 for CH, crude protein, and true protein and 1.9 for energy and NPN to 3.3 for TS. Differences in dispersion between male and female composition were statistically tested by analysis of variance using Levene’s test for all variables that did not exhibit normal distribution and by the F-test for energy, which followed a normal distribution. The variance of all variables (Table 4) was significantly higher in male samples than in female samples, except that of carbohydrate content, for which the difference was marginally rejected (*p* = 0.058).

In transitional BM samples, the Independent Samples *t*-test was only used for the statistical analysis of CH according to the Shapiro–Wilk normality test results presented in Table 1. All other comparisons were performed using the non-parametric Mann–Whitney U test, since at least one group of samples did not pass the Shapiro–Wilk normality test. Contrariwise to our observations in colostrum, the statistical analyses of transitional BM indicated that fat, TS, energy (Table 2), and carbohydrate content (Table 3) exhibited statistically significant higher levels in female samples, with *p*-values well below the threshold of 0.05, whereas crude protein, true protein, and NPN did not show statistically significant differences. The average levels were determined as follows: fat at 2.69 g/dL for males and 4.30 g/dL for females; TS at 13.29 g/dL for males and 15.13 g/dL for females; energy at 66.98 Kcal/dL for males and 83.13 Kcal/dL for females; and CH at 8.37 g/dL for males and 8.84 g/dL for females. Regarding the uniformity within the two groups, similarly, as observed in colostrum, the male transitional BM group exhibited a statistically significant higher variance and, hence, a lower uniformity in fat, TS, and energy content than the female group, as indicated by the respective *p*-values calculated by the F-test (Table 4).

In mature BM, fat and energy sample groups followed a normal distribution and, hence, they were compared using the Independent Samples *t*-test as a parametric statistical method, whereas for the remaining variables, the Mann–Whitney U test as a non-parametric method was chosen. The calculated *p*-values obtained for the *t*-test were 0.815 and 0.934, respectively, indicating no statistically significant differences in the fat and energy content in the mature milk samples from mothers of male and female infants (Table 3). Similarly, no statistically significant differences were identified in the other mature BM variables, analyzed by the Mann–Whitney U test (Table 2). Therefore, our results showed no differences in mature BM with respect to offspring sex.

BM composition seemed to undergo changes throughout the different lactation stages that were not common to both neonatal sexes. To compare the analytical variables across lactation stages, only TS and energy in female births could be analyzed using Welch’s ANOVA, as the Shapiro–Wilk normality test indicated that they were normally distributed and Levene’s test showed unequal variances. In the same groups, post hoc two-way comparisons were performed using the Bonferroni post hoc test. The remaining groups and variables exhibited *p*-values in the Shapiro–Wilk normality test lower than 0.05, indicating a non-normal distribution, and were, therefore, analyzed using the non-parametric Kruskal–Wallis test with post hoc comparisons conducted by the Dunn–Bonferroni method.

The results, presented in Table 5 and illustrated in Figure 2, show that fat levels tended to increase from colostrum to mature BM in both sexes, since the fat content almost doubled in both. The carbohydrate content in female samples remained relatively stable across all stages, as no statistically significant differences were observed between lactation stages, whereas in male samples, colostrum exhibited significantly lower carbohydrate levels, with a mean of 7.22 g/dL compared to 8.37 and 8.42 g/dL in transitional and mature BM, respectively. The nitrogenous compound contents (crude protein, true protein, and NPN) were significantly higher in colostrum than in mature BM for both sexes. Specifically, in male samples, all three analyzed nitrogenous compounds were approximately 2.6 times higher compared to 1.6 times in female colostrum and mature BM samples. This finding is in agreement with the particularly high levels observed in male colostrum, as previously reported. Finally, in female samples, the highest mean values for fat, total solids, and energy were observed in transitional BM, which also showed statistically significant differences compared to both colostrum and mature BM, except for fat, which exhibited no significant difference compared to mature BM.

## 4. Discussion

In the present paper, we report that colostrum samples from mothers of male infants were found to contain higher levels of crude protein and non-protein nitrogen than colostrum samples from mothers of female neonates. However, the concentrations of fat, carbohydrates, and total solids, as well as energy content, showed no statistically significant differences between the sexes. Furthermore, in female transitional BM, the fat, total solids, and energy contents were higher than in male transitional BM, while in mature BM, no statistically significant differences were detected.

Colostrum and transitional milk, despite being produced for relatively short periods, are vital for neonatal development, especially for immune support. Colostrum delivers high levels of immunoglobulins and bioactive compounds that help to compensate for the immature immune system, particularly in preterm infants [25]. Transitional milk continues this immune support with dynamic changes in immunological content tailored to each mother–infant pair, enhancing infection defense and gut microbiome development [26].

The literature on the effects of offspring sex on the composition of BM does not lack contradictions overall. This can be attributed to the lack of standardized methods for human milk collection, variations in study designs, the limited number of studies on this issue, the small sample sizes used in some of the studies, and foremost to the significant variations documented in BM composition according to the circadian cycle, lactation frequency, and within-feeding BM composition variability [27,28]. In the study of Ramiro-Cortijo et al. [29], where no colostrum samples were analyzed, male transitional BM exhibited higher protein and glutathione (GSH) levels, an indicator of antioxidant capacity. In a Brazilian study by Bernardes-Loch et al. [13], sex differences were observed regarding protein content, as well as regarding the proteome in BM across lactation stages.

The Miris Human Milk Analyzer, employing mid-infrared analyses of samples, was selected, as in previous studies [30,31], over the standard AOAC (Association of Official Analytical Chemists) International methods because it requires low volumes and is non-destructive, thus allowing for recovery of the samples. This is particularly important for colostrum, which is produced in small volumes.

According to Alhindi et al. [32], male BM showed a higher protein content than female BM. However, Khelouf et al. [11], in their analysis of 25 male and 16 female colostrum samples, did not identify any differences in BM protein content. Instead, they observed that the fat content of male BM was significantly higher than that in female BM at all lactation stages, while the carbohydrate concentration in transitional milk for female infants was higher than that in milk for male infants. A further study conducted on 189 colostrum samples employing mid-infrared spectroscopy concluded that the macronutrients and energy content in colostrum were not affected by the sex of the offspring [14]. In another study on BM from women in southern Kenya, fat and subsequently energy were found to be higher in female BM than in male BM [33]. Powe et al. [34] reported sex differences in amino acids and particularly higher free glutamine levels in BM for male infants. Similarly, Hosseini et al. [12] reported that Iranian mothers of female infants produced mature milk with a higher fat content compared to mothers of male infants. Another study also found that mature breast milk was higher in carbohydrates and energy for female infants than for male infants [35]. Recently, Wang et al. [36] identified six unique proteins in female BM and nine in male BM, while the abundances of other proteins also varied between the sexes. However, Suwaydi et al. [37] reported that the composition of human milk does not differ based on infant sex. Our results also indicated statistically significant higher fat and energy contents, but only in transitional and not in mature milk.

Lastly, sex differences in non-macronutrient BM components have also been reported. Tonon et al. [38], Wang et al. [39], and Borewicz et al. [40] stated that the types of HMOs are associated with infant sex. Caffé et al. [41] reported sex differences in IgG concentrations, but only in mothers with a low diversity in their diets.

The most profound sex differences reported in the present study concern the nitrogenous compound contents of colostrum, which were nearly 60% higher in the male group than in the female group. In contrast, sex-based differences were more subtle in transitional BM and not statistically significant in mature BM, aligning with the existing literature [42,43]. In addition, Galante et al. [24] reported sex relations with maternal factors in BM growth-related hormones and protein in the Finnish STEPS cohort. The peripartum represents a remarkable phase of endocrine transition. Upon parturition, placenta expulsion causes a rapid drop in sex steroids, exposing the female body to intense hormonal shifts unique to the peripartum phase. As the mother–fetus dyad evolves into the mother–milk–infant triad, the first postpartum days comprise the time when the most acute physiological and biochemical adaptations occur. During pregnancy, male fetal sex is correlated with elevated proinflammatory cytokines and angiogenic factors, whereas female fetal sex is linked to higher regulatory cytokine levels [44]. After birth, the maternal body begins to establish a new hormonal balance, leading to postpartum hormonal fluctuations. These changes may explain the observed differences in nitrogenous compounds and the higher variation in male colostrum samples compared to later-stage BM and the female colostrum values.

There are no available reports on metabolic programming and signaling derived from signaling molecules and sex hormones. Other bioactive components are linked to the relationship between appetite and satiety related to sex or metabolism [45], as well as to the absorption and development of the infant’s gut considering sex. Further research is needed to investigate sex-based differences in a broader range of potentially bioactive components of human milk, such as exosomal miRNAs and the myriad of growth-regulating factors, using rigorous methods and controlling for external confounders, considering maternal weight and nutritional status.

Regarding the changes observed according to lactation stage, our findings are in accordance with the existing literature. Protein content was high in colostrum and decreased with advancing lactation stages, while fat followed the opposite trend. Regarding carbohydrates, no significant differences in female samples were observed, whereas in male samples, colostrum differed significantly from transitional and mature BM. According to the literature, colostrum exhibits lower levels of lactose, by far the most abundant carbohydrate in BM, but is richer in human milk oligosaccharides, which partly compensate for the lower lactose levels [9].

In the present study, significant differences between BM samples from mothers of male and female neonates are reported, probably reflecting distinct nutritional and hormonal requirements corresponding to sex-specific growth and development characteristics. Male infants tend to grow faster, following different growth patterns in terms of weight and length during the first few months of life, whereas female infants may have a slower, steadier growth trajectory [46]. Additionally, males and females may reach developmental milestones at slightly different rates, with evidence showing that females tend to reach neurodevelopmental milestones like walking or talking earlier than male infants [47].

The relatively low number of samples per lactation stage and the sample analysis performed only by mid-infrared spectroscopy constitute limitations of the present study. Furthermore, the relatively high homogeneity of the cohort in terms of racial origin, consisting only of Mediterranean women with similar dietary habits, functions both as a facilitator and a limitation; while a homogeneous cohort creates a smooth, low-noise background that enhances the ability to detect differences compared to a heterogeneous one, it may simultaneously restrict the generalizability of the findings. Hence, cultural factors that typically affect attitudes toward breastfeeding had a limited impact on our findings [48].

## 5. Conclusions

Further research is required in order to elucidate whether the observed differences consist of an evolutionary adaptation to the specific needs of each individual sex or if they result from the biochemical transition from pregnancy to motherhood, particularly in mothers of male infants. The collected samples, alongside the macronutrient analysis by mid infra-red spectrometry presented in this paper, will be submitted to detailed analysis in order to identify more specific possible differences in fatty acid composition, small metabolites, proteins, peptides, and metabolomic and lipidomic profile, as well as hormonal assays that could directly assess endocrine differences in BM. The anticipated findings are expected to provide valuable insights on neonatal feeding requirements with regard to personalized nutritional requirements, opening up future possibilities for tailored neonatal feeding strategies, optimized donor milk formulations, and improved maternal support programs that will contribute to supporting the mother–milk–infant triad.

## Figures and Tables

**Figure 3:**
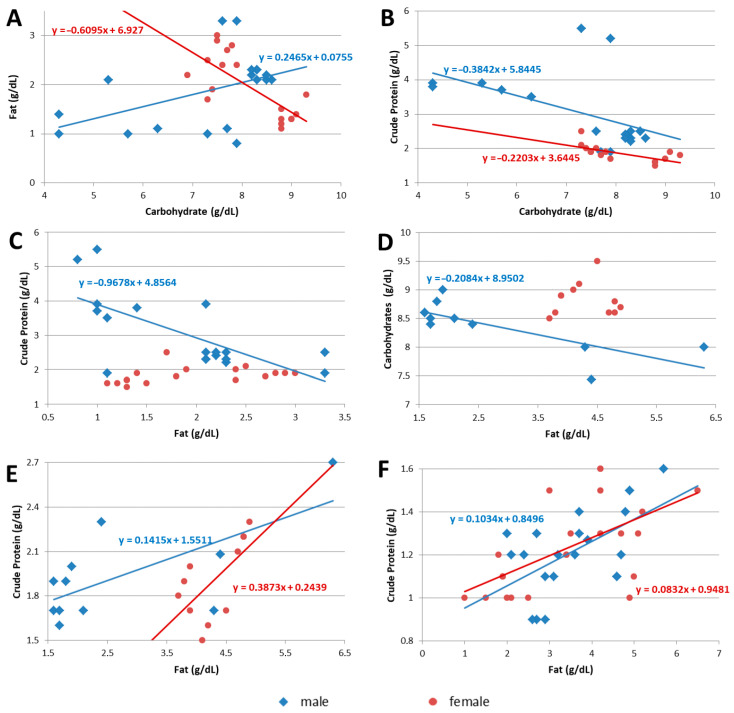
Scatter diagrams of correlated analytes and linear model equations, (**A**) fat to carbohydrate in colostrum, (**B**) crude protein to carbohydrate in colostrum, (**C**) crude protein to fat in colostrum, (**D**) carbohydrate to fat in transitional breast milk, (**E**) crude protein to fat in transitional breast milk, and (**F**) crude protein to fat in mature breast milk. Blue diamonds and red circles represent male and female samples, respectively.

**Table 1:** *p*-Values determined by the Shapiro–Wilk normality test and the statistical method that was used for the comparison of breast milk (BM) of male and female births. Values above 0.05 indicate that the group of analytical values follows normal distribution; thus, univariate analysis between two groups with normal distribution was performed using the Independent Samples *t*-test (*t*-test), whereas in groups not following normal distribution, the Mann–Whitney U test (M-W) was employed. For multiple groups comparison following normal distribution with unequal variances Welch’s Analysis of variance (W-ANOVA) was employed followed by post hoc two-way comparisons by Bonferroni post hoc tests (BPh). Multiple comparisons not following normal distribution were performed by the Kruskal–Wallis test (K-W) and the subsequent post-hoc two-way comparisons by the Dunn–Bonferroni tests (D-B). CH, carbohydrates; NPN, non protein nitrogen; TS, total solids. *p*-values with statistical significance level ≤ 0.05 were added in blue font.

		*p*-Value			Method of Comparison			
		Colostrum	Transitional ΒΜ	Mature ΒΜ	Colostrum	Transitional ΒΜ	Mature ΒΜ	Lactation Stage
Fat	Male	0.022	0.003	0.265	M-W	M-W	*t*-test	K-W/D-B
	Female	0.087	0.150	0.401				K-W/D-B
CH	Male	0.002	0.564	0.001	M-W	*t*-test	M-W	K-W/D-B
	Female	0.027	0.194	0.216				K-W/D-B
Crude Protein	Male	0.019	0.049	0.382	M-W	M-W	M-W	K-W/D-B
	Female	<0.001	0.613	0.021				K-W/D-B
True Protein	Male	0.029	0.026	0.507	M-W	M-W	M-W	K-W/D-B
	Female	<0.001	0.379	0.016				K-W/D-B
NPN	Male	0.149	0.098	0.022	M-W	M-W	M-W	K-W/D-B
	Female	0.002	<0.001	<0.001				K-W/D-B
TS	Male	0.002	0.005	0.011	M-W	M-W	M-W	K-W/D-B
	Female	0.165	0.581	0.677				W-ANOVA/BPh
Energy	Male	0.137	0.011	0.252	*t*-test	M-W	*t*-test	K-W/D-B
	Female	0.108	0.165	0.375				W-ANOVA/BPh

**Table 2:** Key features of the Mann–Whitney U test on the analytical variables not following normal distribution comparing breast milk (BM) samples of male and female births. CH, carbohydrates; NPN, non-protein nitrogen; TS, total solids; SD, standard deviation. *p*-values with statistical significance level ≤ 0.05 were added in blue font.

			*n*	Mean	Minimum	Maximum	Std, Deviation	SD Ratio	Inter- Quartile Range	U	z	Asymptotic *p*	Exact *p*	r
**Colostrum**	Fat	Male	19	1.82	0.8	3.3	0.78	1.2	1.2	134.5	−0.86	0.391	0.397	0.14
		Female	18	1.97	1.1	3.0	0.65		1.1					
	CH	Male	19	7.28	4.3	8.6	1.41	1.8	1.5	103.5	−1.84	0.066	0.066	0.31
		Female	18	8.14	6.9	9.3	0.78		1.3					
	Crude Protein	Male	19	2.96	1.4	5.5	1.12	1.8	1.5	50	−3.55	<0.001	<0.001	0.59
		Female	18	1.97	1.5	4.3	0.63		0.2					
	True Protein	Male	19	2.40	1.1	4.4	0.93	1.8	1.3	56.5	−3.98	<0.001	<0.001	0.62
		Female	18	1.59	1.2	3.5	0.51		0.3					
	NPN	Male	19	0.56	0.2	1.1	0.25	1.9	0.4	86.5	−2.43	0.015	0.016	0.40
		Female	18	0.38	0.2	0.8	0.13		0.1					
	TS	Male	19	12.37	9.4	15	1.93	3.3	3.6	125	−1.16	0.247	0.257	0.19
		Female	18	12.16	11.4	13.6	0.58		0.8					
**Transitional BM**	Fat	Male	12	2.69	1.6	6.3	1.5	3.3	1.25	23	−2.65	0.008	0.007	0.55
		Female	11	4.3	3.7	4.9	0.45		0.85					
	Crude Protein	Male	12	1.99	1.6	2.7	0.37	1.4	0.43	61.5	−0.28	0.78	0.786	0.06
		Female	11	1.91	1.5	2.3	0.27		0.45					
	True Protein	Male	12	1.66	1.4	2.2	0.28	1.0	0.3	55	−0.68	0.496	0.525	0.14
		Female	11	1.54	1.1	1.9	0.27		0.45					
	NPN	Male	12	0.33	0.1	0.5	0.13	1.3	0.11	51.5	−0.94	0.346	0.379	0.2
		Female	11	0.37	0.1	0.5	0.1		0					
	TS	Male	12	13.29	12.1	17.2	1.5	2.5	1.83	19	−2.9	0.004	0.003	0.6
		Female	11	15.13	14	15.9	0.61		0.95					
	Energy	Male	12	66.98	55	102	14.08	2.9	14.68	14	−3.2	0.001	0.001	0.67
		Female	11	83.18	76	89	4.85		9.0					
**Mature BM**	CH	Male	21	8.47	6	9.4	0.82	2.1	0.65	196	−0.85	0.393	0.404	0.13
		Female	22	8.74	7.7	9.4	0.40		0.38					
	Crude Protein	Male	21	1.22	0.9	1.6	0.19	0.9	0.2	227	−0.09	0.932	0.942	0.01
		Female	22	1.24	1	1.6	0.21		0.35					
	True Protein	Male	21	0.98	0.7	1.3	0.14	0.8	0.20	227.5	−0.09	0.931	0.942	0.01
		Female	22	1.00	0.8	1.3	0.18		0.35					
	NPN	Male	21	0.24	0.1	0.4	0.09	1.5	0.1	225	−0.16	0.876	0.894	0.02
		Female	22	0.24	0.2	0.3	0.06		0.1					
	TS	Male	21	13.51	12.1	16.7	1.34	0.8	2.0	226	−0.12	0.903	0.913	0.02
		Female	22	13.63	10.8	17.7	1.72		2.7					

**Table 3:** Key features of the Independent Samples *t*-test on the analytical variables following normal distribution comparing breast milk (BM) samples of male and female births. SD, standard deviation; CH, carbohydrates. *p*-value with statistical significance level ≤ 0.05 was added in blue font.

		Colostrum		Transitional BM		Mature BM			
		Energy		CH		Fat		Energy	
		Male	Female	Male	Female	Male	Female	Male	Female
**Descriptive** **Statistics**	***n***	19	17	12	11	21	22	21	22
	**Mean**	59.21	59.00	8.37	8.84	3.61	3.46	73.25	72.36
	**Minimum**	42	51	7.4	8.5	2	1	59	48
	**Maximum**	72	67	9.0	9.5	5.7	6.5	97	105
	**SD**	9.44	4.92	0.41	0.29	1.07	1.48	10.27	14.89
	**SD ratio**	1.9		1.4		0.7		0.7	
	**Inter-quartile Range**	14	8	0.45	0.35	1.8	2.9	13.0	24.5
***t*-test**	**Mean Difference**	0.21		−0.47		0.14		0.89	
	**Standard Error of Difference**	2.5		0.15		0.39		3.92	
	**Lower limit**	−4.86		−0.78		−0.66		−7.03	
	**Upper limit**	5.28		−0.15		0.94		8.8	
	**t**	0.09		−3.1		0.36		0.23	
	***df***	27.42		21		41		41	
	***p***	0.932		0.010		0.722		0.822	

**Table 4:** Key features of analysis of variance employing the F-test for normal and the Levene’s test for non-normal distribution and ratio of the standard deviations of all male–female sample comparisons. BM, breast milk; CH, carbohydrates; NPN, non-protein nitrogen; TS, total solids. *p*-values with statistical significance level ≤ 0.05 were added in blue font.

			SD Ratio	*p*-Value	F
**F-test**	Colostrum	Energy	1.9	0.010	3.68
	Transitional BM	CH	1.4	<0.001	0.054
	Mature BM	Fat	0.7	<0.001	0.028
		Energy	0.7	0.870	0.930
**Levene’s test**	Colostrum	Fat	1.2	0.404	0.71
		CH	1.8	0.058	3.84
		Crude Protein	1.8	0.003	10.34
		True Protein	1.8	0.001	12.56
		NPN	1.9	0.008	7.87
		TS	3.3	<0.001	51.25
	Transitional BM	Fat	3.3	0.012	7.59
		Crude Protein	1.4	0.419	0.68
		True Protein	1.0	0.887	0.02
		NPN	1.3	0.298	1.14
		TS	2.5	0.032	5.25
		Energy	2.9	0.025	5.79
	Mature BM	CH	2.1	0.072	3.4
		Crude Protein	0.9	0.440	0.61
		True Protein	0.8	0.108	2.69
		NPN	1.5	0.033	4.88
		TS	0.8	0.059	3.76

**Table 5:** Comparison of the characteristics of different stages of human breast milk (BM), colostrum, transitional, and mature BM. n.a.: not applied. *p*-values with statistical significance level ≤ 0.05 were added in blue font.

		Overall *p*-Value			Descriptive Statistics				Inferential Statistics					
			Colostrum		Transitional		Mature		Colostrum–Transitional		Colostrum–Mature		Transitional–Mature	
			*n*	Mean	*n*	Mean	*n*	Mean	*p*- Value	Std. Test Statistic	*p*- Value	Std. Test Statistic	*p*- Value	Std. Test Statistic
**Male**	Fat	<0.001	19	1.82	12	2.69	22	3.61	0.532	−1.35	<0.001	−4.5	0.031	−2.56
	CH	<0.001	19	7.28	12	8.37	22	8.47	0.052	−2.38	<0.001	−3.94	0.917	−1.02
	Crude Protein	<0.001	19	2.96	12	1.99	22	1.22	0.288	1.66	<0.001	6.1	0.001	3.64
	True Protein	<0.001	19	2.40	12	1.66	22	0.98	0.411	1.49	<0.001	6.04	<0.001	3.77
	NPN	<0.001	19	0.56	12	0.33	22	0.24	0.060	2.33	<0.001	4.79	0.206	1.82
	TS	0.445	19	12.37	12	13.29	22	13.51	n.a.	n.a.	n.a.	n.a.	n.a.	n.a.
	Energy	0.001	19	59.21	12	66.98	22	73.25	0.779	−1.13	0.001	−3.6	0.137	−2.00
**Female**	Fat	<0.001	18	1.97	11	4.3	21	3.46	<0.001	−4.33	0.002	−3.4	0.358	1.56
	CH	0.081	18	8.14	11	8.84	21	8.74	n.a.	n.a.	n.a.	n.a.	n.a.	n.a.
	Crude Protein	<0.001	18	1.97	11	1.91	21	1.24	1	−0.29	<0.001	5.1	<0.001	1
	True Protein	<0.001	18	1.59	11	1.54	21	1.00	1	−0.15	<0.001	4.97	<0.001	1
	NPN	<0.001	18	0.38	11	0.37	21	0.24	1	−0.37	<0.001	4.04	<0.001	1
	TS	<0.001	18	12.16	11	15.13	21	13.63	<0.001	−6.35 *	0.001	−3.78 *	0.005	3.33 *
	Energy	<0.001	18	59.00	11	83.18	21	72.36	<0.001	−6.01 *	0.001	−4 *	0.023	2.79 *

* The *t*-value is given as a measure of the Standard Test Statistic. The pairwise correlations of the major primary analytes were investigated within each sample, grouped by sex and lactation stage (Table 6 and Figure 3). Of the nine possible combinations in each sex, statistical significance was demonstrated in six correlations among male samples and in four among females. The macronutrients in colostrum showed stronger correlations with each other, exhibiting five statistically significant correlations out of six possible correlations tested. Most correlations were strong, with coefficients reaching −0.76 and −0.73 between the fat and carbohydrate contents in female colostrum and transitional BM, respectively. Fat and crude protein were positively correlated in both sexes in transitional and mature BM, but negatively correlated in male colostrum samples, further underscoring differences and the unique characteristics of colostrum compared to later-stage BM.

**Table 6:** Pairwise correlation coefficient (r) and *p*-values of macronutrients in male and female samples. Carbohydrates: CH; crude protein: CPr. *p*-values with statistical significance level ≤ 0.05 were added in blue font.

		Male		Female	
		r	*p*-Value	r	*p*-Value
**Colostrum**	CH and CPr	−0.51	0.029	−0.68	0.002
	FAT and CH	0.47	0.050	−0.73	0.001
	FAT and CPr	−0.68	0.002	0.23	0.368
**Transitional breast milk**	CH and CPr	−0.33	0.297	−0.55	0.078
	FAT and CH	−0.76	0.007	0.02	0.947
	FAT and CPr	0.68	0.022	0.65	0.031
**Mature breast milk**	CH and CPr	0.24	0.289	0.06	0.788
	FAT and CH	−0.15	0.514	0.22	0.327
	FAT and CPr	0.57	0.007	0.59	0.004

## Data Availability

Data described in the manuscript will be made available upon request pending application and approval. The data are not publicly available due to the ongoing nature of the study.

## Supplementary Materials

The following supporting information can be downloaded at: https://www.mdpi.com/article/10.3390/nu17091422/s1. Table S1: Descriptive statistics of demographic and Clinical Characteristics of Mothers and Neonates (n = 36) in the Colostrum Group; Table S2: Descriptive statistics of demographic and Clinical Characteristics of Mothers and Neonates (n = 23) in the Transition Milk Group; Table S3: Descriptive statistics of demographic and Clinical Characteristics of Mothers and Neonates (n = 43) in the Mature Milk Group.

## Abbreviations

The following abbreviations are used in this manuscript:

BM	Breast milk
CH	Carbohydrate
HMOs	Human milk oligosaccharides
IQR	Interquartile range
NPN	Non-protein nitrogen
SD	Standard deviation
TS	Total solids
