# Supplementary material for: From Mother–Fetus Dyad to Mother–Milk–Infant Triad: Sex Differences in Macronutrient Composition of Breast Milk
Source: Nutrients. 2025 Apr 23;17(9):1422. doi: 10.3390/nu17091422 (PMC12073663; doi:10.3390/nu17091422)
Supplement: Supplementary file 1 [file nutrients-17-01422-s001.zip › nutrients-3587212-supplementary.pdf]

From Mother-Fetus Dyad to Mother-Milk-Infant Triad: Sex Differences in the Macronutrient Composition of Breast Milk

Maria Lithoxopoulou [1], Calliope Karastogiannidou [2], Anastasia Karagkiozi [3], Iliani Eleni Zafeiriadou [2], Ekaterini Pilati [2], Elisavet Diamanti [1*], Stavros Kalogiannis [2‡*], Emilia Vassilopoulou [2,4,5,6‡]

Supplementary Materials

Table S1: Descriptive statistics of demographic and Clinical Characteristics of Mothers and Neonates (n=36) in the Colostrum Group

Infant's sex<br>Male n= 19<br>Female n= 17	**n**	**Mean**	**Minimum**	**Maximum**	**Std. Deviation**	**SD ratio**
Maternal age (years)	36	31.7	21.0	42.0	4.9	15.6
Paternal age (years)	36	35.6	26.0	46.0	5.1	14.3
Maternal BMI (kg/m2)	36	24.2	17.7	33.2	4.0	16.5
Number of birth	28	2.0	1.0	4.0	0.8	42.7
Normal delivery	19	-	-	-	-	-
Cesarean section	9	-	-	-	-	-
Pre-pregnancy weight (kg)	36	66.4	47.0	90.0	11.2	16.9
Pregnancy weight gain (kg)	28	12.9	5.0	27.0	5.0	38.9
Gestational age (weeks)	36	38.4	37.0	40.0	1.0	2.5
Infant birth weight (g)	36	3247.2	2450.0	4080.0	382.9	11.8
Infant weight at discharge (g)	27	3072.6	2480.0	3760.0	367.1	11.9
Infant weight loss in clinic (%)	23	6.0	1.2	10.9	2.7	45.0
Duration of breastfeeding previous children (months)	19	13.5	2.0	30.0	7.0	51.5

Table S2: Descriptive statistics of demographic and Clinical Characteristics of Mothers and Neonates (n=23) in the Transition Milk Group.

Infant's sex<br>Male n=12<br>Female n=11	**n**	**Mean**	**Minimum**	**Maximum**	**Std. Deviation**	**SD ratio**
Maternal age (years)	23	30.4	23.0	38.0	4.5	14.7
Paternal age (years)	23	35.5	26.0	46.0	5.2	14.7
Maternal BMI (kg/m2)	23	24.0	17.7	31.2	3.5	14.5
Number of birth	23	2.0	1.0	3.0	0.6	32.6
Normal delivery	15	-	-	-	-	-
Cesarean section	8	-	-	-	-	-
Pre-pregnancy weight (kg)	23	66.5	46.0	88.0	10.7	16.0
Pregnancy weight gain (kg)	23	12.7	5.0	24.0	4.8	37.9
Gestational age (weeks)	23	38.6	37.0	40	0.9	2.3
Infant birth weight (g)	23	3381.7	2660.0	4500.0	468.6	13.9
Infant weight at discharge (g)	23	3182.2	2500.0	4370.0	463.6	14.6
Infant weight loss in clinic (%)	21	6.5	1.2	10.9	2.7	41.3
Duration of breastfeeding previous children (months)	17	11.7	2.0	30.0	7.4	63.0

Table S3: Descriptive statistics of demographic and Clinical Characteristics of Mothers and Neonates (n=43) in the Mature Milk Group.

Infant's sex<br>Male n=22<br>Female n=21	**n**	**Mean**	**Minimum**	**Maximum**	**Std. Deviation**	**SD ratio**
Maternal age (years)	43	31.4	23.0	42.0	5.1	16.1
Paternal age (years)	36	35.6	26.0	46.0	5.0	14.2
Maternal BMI (kg/m2)	43	23.4	17.7	31.2	3.2	13.9
Number of birth	43	2.0	1.0	4.0	0.8	40.8
Normal delivery	25	-	-	-	-	-
Cesarean section	12	-	-	-	-	-
Pre-pregnancy weight (kg)	43	64.8	46.0	88.0	9.9	15.3
Pregnancy weight gain (kg)	43	12.5	5.0	27.0	4.9	39.2
Gestational age (weeks)	43	38.5	37.0	40.0	0.9	2.3
Infant birth weight (g)	43	3,288.9	2,660.0	4,500.0	413.9	12.6
Infant weight at discharge (g)	36	3,110.8	2,480.0	4,370.0	429.5	13.8
Infant weight loss in clinic (%)	32	6.2	1.2	11.1	2.6	41.0
Duration of breastfeeding previous children (months)	26	13.3	2.0	30.0	6.9	51.7
